# First Report of *Sarcocystis Masoni* in a Captive Alpaca (*Vicugna Pacos*) From China

**DOI:** 10.3389/fvets.2021.759252

**Published:** 2021-10-14

**Authors:** Nan Jiang, Shilin Xin, Niuping Zhu, Liulu Yang, Wei Huang, Junjie Hu, Xiuhong Zhu, Yurong Yang

**Affiliations:** ^1^College of Veterinary Medicine, Henan Agricultural University, Zhengzhou, China; ^2^School of Biological Sciences, Yunnan University, Kunming, China; ^3^College of Forestry, Henan Agricultural University, Zhengzhou, China

**Keywords:** *Sarcocystis* spp., alpaca, parasite load, food safety, China

## Abstract

**Background:** Sarcocystosis is a parasitic disease caused by intracellular protozoan parasite of the genus *Sarcocystis*. Tissue samples of alpacas (*n* = 4) from Henan province (China) were screened for *Sarcocystis* spp. infection by histological examination, pepsin digestion, and molecular assays.

**Results:** *Sarcocystis* spp. was detected in heart, liver, spleen, lung, and kidney of an alpaca by molecular assays. Many sarcocysts with inflammation responses were observed in this alpaca myocardium, and they showed a high similarity to *Sarcocystis masoni* by sequence analysis.

**Conclusion:** This study is the first to demonstrate *Sarcocystis* spp. infection in alpaca from China. The higher parasite load in the alpaca myocardium indicated that it had contact with an environment contaminated with sporocysts, and that the alpaca was susceptible to *Sarcocystis* spp.

## Introduction

Sarcocystosis is a parasitic disease caused by intracellular protozoan parasite of the genus *Sarcocystis*. Its hosts include intermediate and definitive hosts. Intermediate hosts (birds, mammals, reptiles) are infected by ingesting sporozoites shed by a definitive host (typically carnivores) (1). More than 200 recognized *Sarcocystis* species worldwide can infect domestic and wild animals, causing a significant adverse impact on food health and economic development (1, 2).

According to morphological and molecular characteristics, there are two kinds of *Sarcocystis* spp. in alpaca: macroscopic (the macrocysts of *S. aucheniae*) and microscopic (the microcysts of *S. masoni*) (3–7). There have been many reports about *Sarcocystis* spp. infection in the alpacas from Africa and South America (1, 8–10). However, there is no report of *Sarcocystis* spp. infection in alpacas from China.

Most animals infected with *Sarcocystis* spp. have no clinical symptoms, except in the cases of high doses of infection or immunodeficiency of the host, which result in anorexia, fever, anemia, weakness, weight loss, neurological symptoms, diarrhea, and death (6, 7, 11). Gastroenteritis, nausea, diarrhea, colic, shivering, and breathing problems can occur in humans after eating raw or uncooked meat that contains some species of *Sarcocystis*, for example *S. hommins* or *S. suihominnis* (1). In this article, *S. masoni* was detected in the myocardium of an alpaca. This is the first report of a *Sarcocystis* spp. infection in alpaca from China.

## Materials and Methods

### Naturally Infected Alpacas and Sampling

Four dead alpacas (*Vicugna pacos*) from a zoo were collected in Zhengzhou city, Henan province (33°N, 113.30°E), China. Both of them were born in this zoo.

Case 1 was a 4-year-old female, breastfeeding a 3-month-old pup, displaying skin phyma and dyspnea, which suddenly died on June 20, 2020. Case 2 was an adult female with a systemic abscess and a stillborn fetus 1 month before death, which then showed neurological signs and died on August 6, 2020. Case 3 was a 1-year-old male with dyspnea and fever and died on June 4, 2021. Case 4 was an adult male, also displaying dyspnea and died on June 7, 2021. Treatments included azithromycin and multi-vitamins, which alleviated their clinical symptoms. However, the animals ultimately died. The tissues of case 1 (spleen, liver, lung, heart, kidney, or skin phyma), case 2 (kidney and brain), case 3 (blood, spleen, liver, lung, heart, kidney, and lymphonodus), and case 4 (blood, spleen, liver, lung, heart, kidney, intestine, and lymphonodus) were sent to the pathology laboratory of Henan Agricultural University for pathological diagnosis. These samples were also screened for *T. gondii* and *Sarcocystis* spp. infection. However, they all showed negative results for *T. gondii* by histological examination, pepsin digestion, and molecular assays. By serological assays, only case 3 showed anti-*T. gondii* antibody (modified agglutination test: 1:50 titer).

### Light Microscopy Examination

The squashed myocardium sections of alpacas were directly examined for *Sarcocystis* spp. If sarcocysts were found, single cyst was separated from the slide. Three tissue pieces from each sample were processed using conventional paraffin histological methods, and 5-μm-thick sections were prepared and stained with hematoxylin and eosin. The tissue sections were examined for the presence of *Sarcocystis* spp. under a light microscope (Olympus Bx43, Tokyo, Japan).

### Pepsin Digestion Examination

Myocardium (50 g) samples were homogenized and digested in acidic pepsin individually (12). The digested tissues were examined for *Sarcocystis* bradyzoites and cysts by light microscopy.

### Molecular Analysis of *Sarcocystis*

DNA was extracted from the heart, liver, spleen, lung, kidney, brain, lymphonodus, intestine, skin phyma, myocardium digestive juices, and single cyst from the alpacas using a commercial DNA extraction kit (DP304; Tiangen Biotec Company, Beijing, China). PCR amplification of a 900-bp 18S rDNA segment was performed using the primer pair SarcoFext (GGTGATTCATAGTAACCGAACG) and SarcoRext (GATTTCTCATAAGGTGCAGGAG) to check the *Sarcocystis* spp. infection in alpacas (13). In addition, PCR was performed to amplify a segment of the mitochondrial cytochrome *c* oxidase subunit 1 gene (cox 1) using the specific primer pairs of SF1 (ATGGCGTACAACAATCATAAAGAA) and SR9 (ATATCCATACCRCCATTGCCCAT). The amplified PCR product was approximately 1038 bp (14, 15), and both negative and positive controls were included. The PCR products were sent to Shanghai Sangon Company (China) for bi-directional sequencing on an ABI PRISM™ 3730 XL DNA Analyzer using the BigDye Terminator v3.1 Cycle Sequencing Kit (Applied Biosystems, Foster City, CA, USA). The obtained sequences were analyzed by BLAST (GenBank database). The phylogenetic tree of the *Sarcocystis* spp. was constructed using the neighbor-joining method (Kimura two-parameter model).

## Results

### Pathological Findings

**Figure 1:**
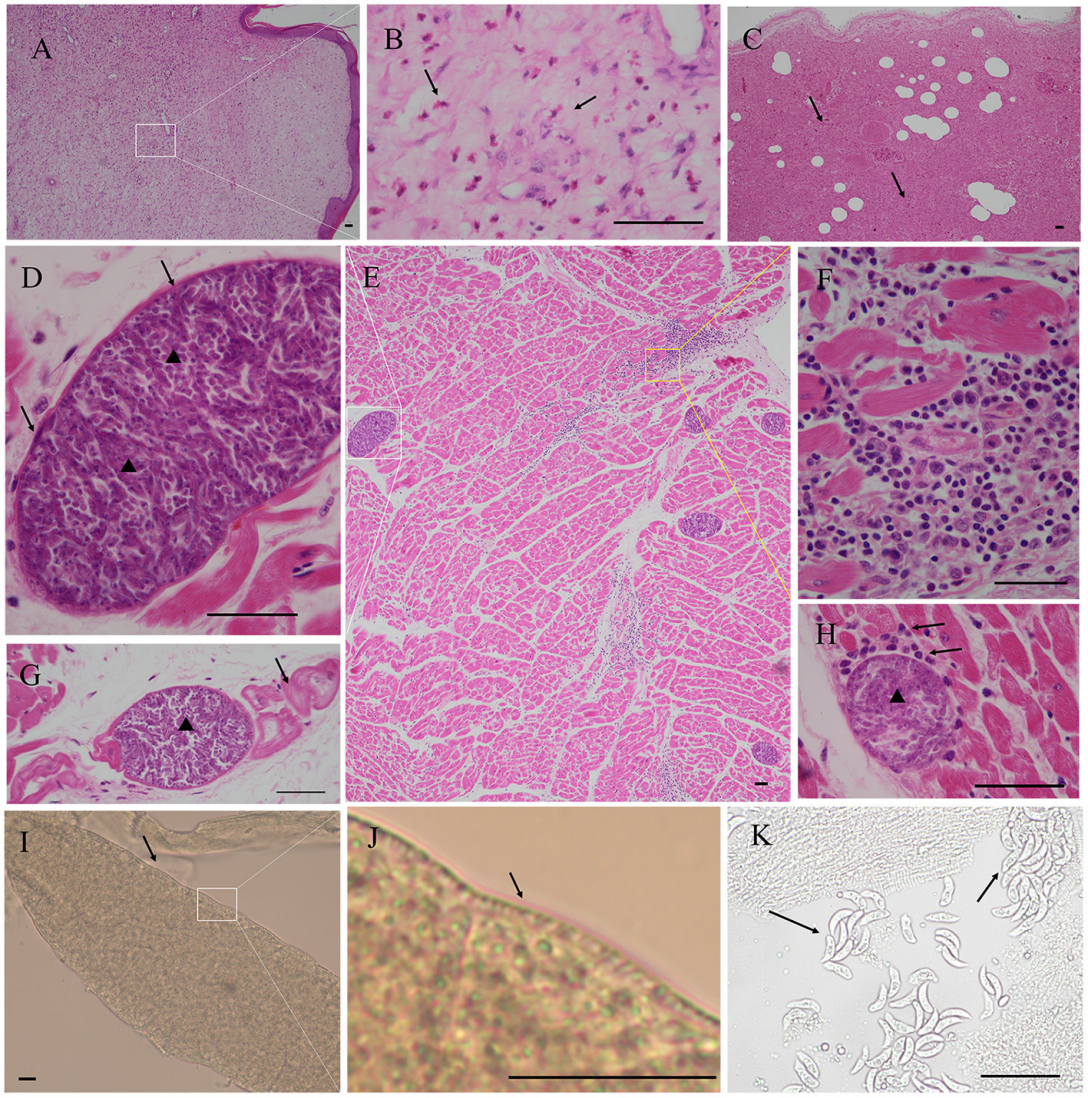
Pathological findings in the myocardium of case 1 alpaca (*Vicugna pacos*). **(A)** Granulation tissue was observed in skin phyma. **(B)** Magnification of picture A; note the presence of numerous eosinophils (arrow), skin phyma. **(C)** Severe interstitial pneumonia, necrosis, and exudation were observed in the lung (arrow). **(D)** Magnification of picture E; *Sarcocystis masoni* cysts were observed in the myocardium, the sarcocyst was separated by septa and formed several rooms, and the bradyzoites were arranged in packets (arrowhead), thin wall (arrow). **(E)** Presence of numerous *S. masoni* cysts in the myocardium. **(F)** Magnification of picture E, myocardial necrosis, and many lymphocytes, macrophages, and plasma cell infiltration. **(G)** *S. masoni* (arrowhead) in Purkinje fibers (arrow). **(H)** Inflammatory responses (arrow) around the degenerated *S. masoni* sarcocyst (arrowhead) in the myocardium and mononuclear cell infiltration (arrow). **(I)** *S. masoni* sarcocyst was found in the pepsin digestion liquid. Thin wall, unstained. **(J)** Magnification of picture I; short and scattered villar protrusions were visible. **(K)** S. *masoni* bradyzoites in the pepsin digestion liquid, unstained. Hematoxylin-eosin **(H, E)** staining; bar = 50 μm.

No visible macroscopic sarcocyst was found in the tissues of four alpacas. Acute interstitial pneumonia, septicemia of the spleen, necrosis of hepatocytes, lipofuscin deposition, renal interstitial hemorrhage, acute glomerulonephritis, a large area of congestion in the lungs, and a large number of inflammatory cell infiltration in the myocardium were observed in case 1. Many fusiform or oval sarcocysts were observed in the myocardium and Purkinje fibers. An obvious inflammatory response (lymphocytes, macrophages, and plasma cell infiltration) was observed in the myocardium. Granulation tissue was observed in skin phyma. Pathological results showed that the infection of the *Sarcocystis* spp. is not the direct cause of the death of this alpaca, but respiratory failure due to acute interstitial pneumonia (Figure 1).

The average sarcocysts load was 79.00±4.58 per square centimeter in the myocardium. The cysts size was about 36.48–204.41 × 31.32–102.01 μm (*n* = 70). The cysts can be seen on the smear after digestion by pepsin. The cysts wall was about 2.65±1.35 μm thick (*n* = 25), and short and scattered villar protrusions were visible. The size of the bradyzoites was about 14–20 × 4–5 μm (*n* = 10) (Figure 1).

For case 2, tissues (kidney and brain) were stored at −20°C for 2 weeks before being sent to the pathology laboratory of Henan Agricultural University. Thus, detailed pathological information could not be obtained. Both case 3 and case 4 showed acute interstitial pneumonia.

### PCR Identification of *Sarcocystis* spp. Infection

**Figure 2:**
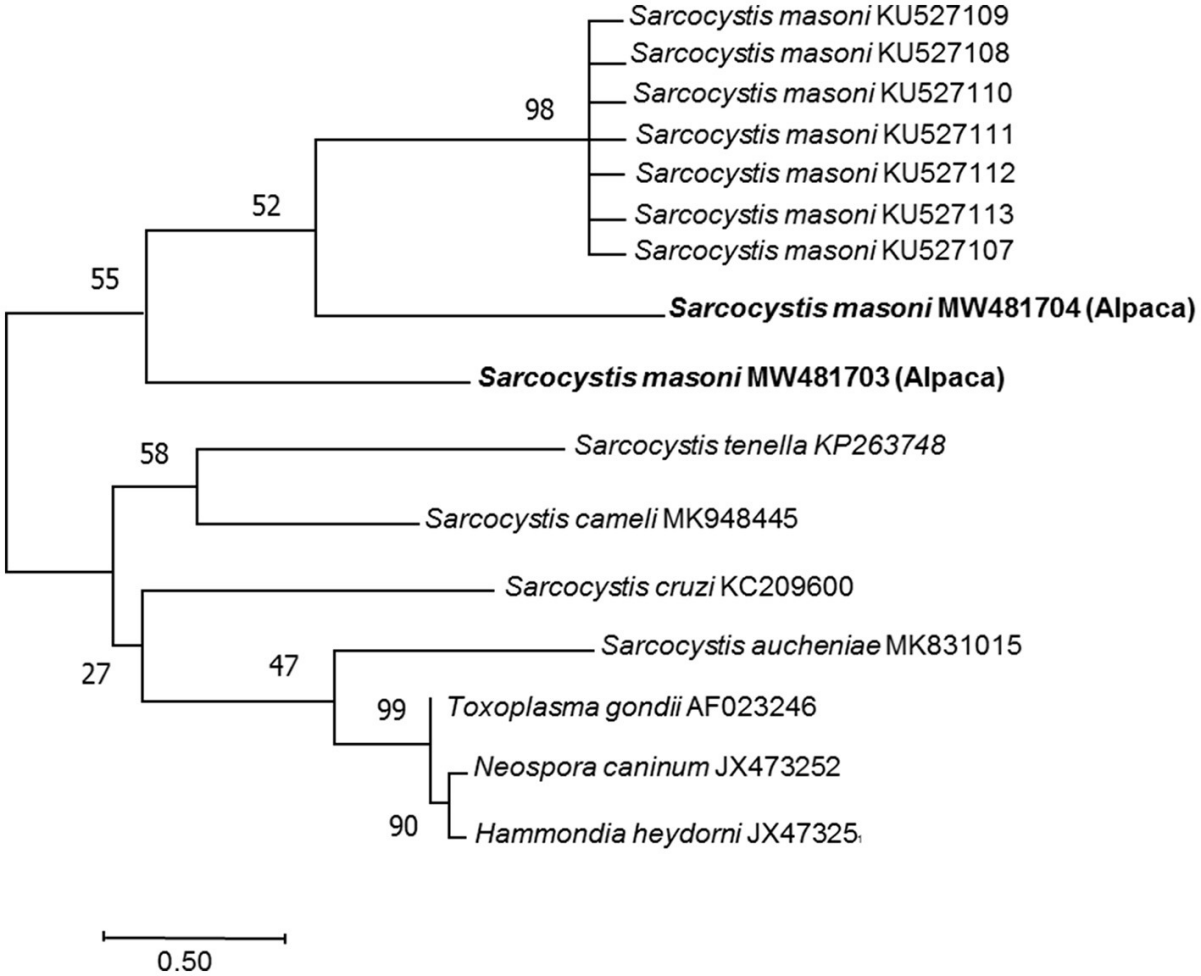
Phylogenetic tree among the *Sarcocystis masoni* from the alpaca identified in this study and other *Sarcocystis* spp. The phylogeny was inferred from the neighbor-joining analysis of the 18S rRNA sequences based on the distances calculated using the Kimura two-parameter model. Bootstrap values *N* > 50% from 1,000 replicates are shown at the nodes. *Sarcocystis* spp. (MW481703, MW481704) close relationship to *Sarcocystis masoni* (KU527107, KU527108, KU527109, KU527110, KU527111, KU527112, and KU527113).

DNA from the samples of heart, liver, spleen, lung, kidney, single cysts, and pepsin-digested myocardium of case 1 had amplified fragments of approximately 900 bp by primers of 18S rRNA (Supplementary Figure). By primers of cox1, except kidney, fragments of 1,038 bp was also amplified from these samples (Supplementary Figure), indicating the presence of *Sarcocystis* spp. The DNA from the samples of case 2, case 3, and case 4 was negative for *Sarcocystis* spp. Sequence analysis was performed on the PCR products from heart, liver, spleen, single cyst, and digestive fluid of case 1. The nucleotide sequences from the sarcocysts of the alpaca were submitted to GenBank (for 18S rRNA: accession number MW481703, MW481704, for cox 1: accession number PRJNA759055). BLAST results showed that the most similar sequences for MW481703, MW481704 in GenBank were *S. masoni* KU527113.1, KU527112.1, KU527111.1, KU527110.1, KU527109.1, and KU527108.1 (100% identity) (Figure 2). PRJNA759055 sequence had similarity to *S. rommeli* hosted by cattle, accession numbers KY120286.1, with only 87% pairwise identity.

## Discussion

*Sarcocystis* infection is common in many species of animals worldwide, including China. Sarcocysts could be found in skeletal muscles and myocardium (1, 16), smooth muscles, and the central nervous system (1, 17). They have an adverse effect on the livestock industry, food health, and animal reproduction. However, there is no vaccine to protect animals against clinical sarcocystosis.

Studies have reported antibodies against *T. gondii* in 30% and 44% of llamas from Peru and the USA (18–20) and in 9% of alpacas from China (21). In this study, antibodies to *T. gondii* (modified agglutination test: 1:50 titer) were found in case 3 alpaca.

Alpacas are raised on a large scale in South America, Africa, Australia, Europe and the United States. Their meat and wool have economic value (10, 22, 23). Macroscopic (*S. aucheniae*) and microscopic (*S. masoni*) sarcocysts were identified in alpacas. Canids are potential definitive hosts (1, 5). *S. aucheniae* appeared in skeletal muscles, and *S. masoni* were commonly found in the cardiac muscle of alpaca. However, little is known of clinical sarcocystosis in alpaca. Severe myositis was reported in an alpaca (6). Human consumption of *Sarcocystis*-infected meat (raw or undercooked beef or pork) may lead to gastroenteritis and diarrhea (1, 22).

Compared to 18S rRNA, cox 1 was found better in differentiation of closely related but morphologically indistinguishable *Sarcocystis* spp. from cattle (24, 25). In this study, BLAST results showed that the most similar sequences for *Sarcocystis* spp. from alpaca were *S. masoni* (100% identity) by 18S rRNA and *S. rommeli* (87% identity) by cox 1 gene. Further, recent research found that cox 1 gene from camels in Saudi Arabia showed low similarity to two *Sarcocystis* spp. (*S. aucheniae* or *S. masoni*) from camels and suggests identification of *Sarcocystis* spp. with more taxa and different molecular markers, including 18S rRNA and cox 1 (15). Mohamed et al. (26) also suggested using the hybrid approach to characterize the microbe, including Next-Generation Sequencing and conventional PCR combined with Sanger sequencing, and also using RFLP-PCR for characterization of *Sarcocystis* isolated from camel (27).

Alpacas in China are mainly imported from Australia. Then, most of them are bred in zoos, while some are kept at home as pets. In this study, all the four cases of alpacas were bred in zoo. According to the sarcocyst's morphology, location, host, and molecular characteristics, the sarcocysts in cardiac muscle of alpaca maybe *S. masoni*. *S. masoni* nucleotides (18S rRNA) were amplified in the heart, liver, spleen, lung, and kidney of the case 1 alpaca (Supplementary Figure). This indicated that except for the striated muscles, *S. masoni* could grow or exist in other tissues, providing molecular evidence of the *S. masoni* infection in other tissues. However, the life cycle of *S. masoni* in the intermediate host and definitive host is unknown.

Both case 1 and case 2 alpacas showed skin granulation tissue or systemic abscess. These findings agree with a previous report of an alpaca with a case history of multiple subcutaneous abscesses (6). More investigation is needed to confirm whether sarcocystosis is associated with multiple abscesses in alpacas.

Although parasite loads from different hosts are not comparable. The average myocardial sarcocyst load in alpaca was 79.00 per square centimeter, which is much higher than in beef (1.40 cysts/cm^2^) and feral pigs (3.03 cysts/cm^2^) from the United States and mutton (7.84 cysts/cm^2^), sheep (18.07 cysts/cm^2^), and cattle (7.1 cysts/cm^2^) from China (28–32). In this study, the higher parasite load in the alpaca indicated that it had contact with an environment contaminated with sporocysts, and that the alpaca was susceptible to *S. masoni*. Poor sanitation and contact with dogs are considered risk factors for *Sarcocystis* spp. infection in alpacas (10). For alpaca meat, boiling (100°C for 10 min), baking (105°C for 65 min), and freezing (−20°C for 10 days) could inactivate the *Sarcocystis* spp. and reduce its risk for other animals (33). This work is the first report of *Sarcocystis* spp. infection in alpaca from China. It is necessary to further explore the natural clinical cases of sarcocystosis to determine the infection source and the definitive host.

## Conclusion

This study is the first to demonstrate *Sarcocystis* spp. infection in alpaca from China. The higher parasite load in the alpaca myocardium indicated that it had contact with an environment contaminated with sporocysts, and that the alpaca was susceptible to *Sarcocystis* spp. Further study will focus the possible route of infection of these animals, ultrastructure of the cysts (walls and protrusions), and molecular characteristics with more markers of these parasites.

## Data Availability Statement

The datasets presented in this study can be found in online repositories. The names of the repository/repositories and accession number(s) can be found below: https://www.ncbi.nlm.nih.gov/genbank/, MW481703, MW481704.

## Ethics Statement

The animal study was reviewed and approved by the Institutional Animal Use Protocol Committee of the Henan Agricultural University, China.

## Author Contributions

NJ performed the data analysis and wrote the manuscript. SX, NZ, LY, and WH helped in collecting samples. JH and XZ helped in the revision of the manuscript. YY designed the experiment and wrote the manuscript. All authors have read and approved the final version of the manuscript.

## Funding

This study was financed by the Natural Science Foundation of Henan Province, China (202300410214) and the Key research projects of Henan higher education institutions (21A230009).

## Conflict of Interest

The authors declare that the research was conducted in the absence of any commercial or financial relationships that could be construed as a potential conflict of interest.

## Publisher's Note

All claims expressed in this article are solely those of the authors and do not necessarily represent those of their affiliated organizations, or those of the publisher, the editors and the reviewers. Any product that may be evaluated in this article, or claim that may be made by its manufacturer, is not guaranteed or endorsed by the publisher.
