# Supplementary material for: First Report of Sarcocystis Masoni in a Captive Alpaca (Vicugna Pacos) From China
Source: Front Vet Sci. 2021 Oct 14;8:759252. doi: 10.3389/fvets.2021.759252 (PMC8551384; doi:10.3389/fvets.2021.759252)
Supplement: Supplementary file 1 [file Image_1.pdf]

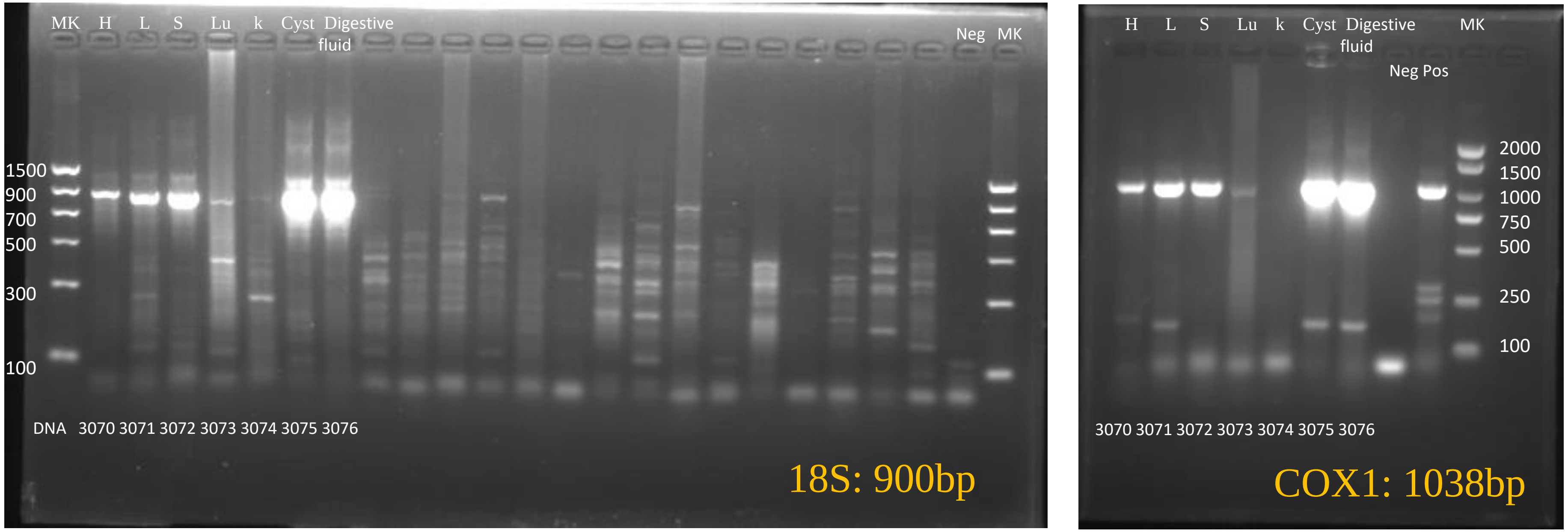

supplement Figure Gel electrophoresis of *Sarcocystis* spp. PCR amplification results from tissues of Alpaca#1

Left figure-by primers 18S rRNA; right figure-by primers cox 1.

3070- Heart; 3071-Liver; 3072-Spleen; 3073-Lung; 3074-Kidney; 3075-Single cyst;
3076-striated muscle digestive fluid; Neg-Negtive control; Pos-positive control; MK-Markers.
